# Reduction in Serum Concentrations of Uremic Toxins Driven by *Bifidobacterium Longum* Subsp.* Longum* BL21 is Associated with Gut Microbiota Changes in a Rat Model of Chronic Kidney Disease

**DOI:** 10.1007/s12602-024-10293-5

**Published:** 2024-06-03

**Authors:** Yao Dong, Zhonghui Gai, Mei Han, Jiaqi Xu, Kang Zou

**Affiliations:** 1 Germline Stem Cells and Microenvironment Lab, College of Animal Science and Technology, Nanjing Agricultural University, Nanjing, 210095 China; 2 Stem Cell Research and Translation Center, Nanjing Agricultural University, Nanjing, 210095 China; 3 Department of Research and Development, Wecare Probiotics Co., Ltd, Suzhou, 215200 China; 4 Department of Food Quality and Safety, Shanghai Business School, Shanghai, 200235 China

**Keywords:** Chronic kidney disease, Gut microbiota dysbiosis, Uremic toxins, Probiotic intervention, *Bifidobacterium longum* subsp.* longum*

## Abstract

Gut microbiota dysbiosis and consequent impairment of gut barrier function, culminating in elevated levels of uremic toxins, are prevalent in chronic kidney disease (CKD) patients. These toxins, notably indoxyl sulphate (IS), indole-3-acetic acid (IAA), and trimethylamine oxide (TMAO), are implicated in a spectrum of CKD-related complications, including cardiovascular disease, bone and mineral disorders, and inflammation. The specific impacts of various probiotics on these CKD manifestations remain unexplored. This study delved into the potential of dietary probiotic interventions, particularly *Bifidobacterium longum* subsp. *longum* BL21, to modulate gut microbiota and mitigate metabolic disorders in a CKD rat model. Over a six-week period, we administered a dietary regimen of BL21 and conducted comprehensive analyses, including serum uremic toxin quantification and 16S rRNA gene sequencing, to systematically profile gut microbial alterations at the phylogenetic level. Our findings reveal that BL21 intervention significantly ameliorated CKD-induced disruptions in gut microbial populations, enhancing both microbial richness and the relative abundance of key taxa. Importantly, BL21 appeared to exert its beneficial effects by modulating the abundance of crucial species such as *Barnesiella* and *Helicobacter*. Functionally, the intervention markedly normalized serum levels of IS, IAA, and TMAO, while potentially attenuating p-cresol sulphate (PCS) and p-cresol glucuronide (PCG) concentrations. Consequently, BL21 demonstrated efficacy in regulating gut microbiota and curtailing the accumulation of uremic toxins. Our results advocate for the utilization of BL21 as a dietary intervention to diminish serum uremic toxins and re-establish gut microbiota equilibrium at the phylogenetic level, underscoring the promise of probiotic strategies in the management of CKD.

**Supplementary Information:**

The online version contains supplementary material available at 10.1007/s12602-024-10293-5.

## Introduction

Chronic kidney disease (CKD) poses a growing global health challenge, with its rising incidence and significant contribution to cardiovascular morbidity and mortality [1]. The gut microbiota has emerged as a critical player in CKD progression, implicated in the genesis of uremic toxins and systemic inflammation [2, 3]. Uremic toxins such as indoxyl sulphate (IS), p-cresol glucuronide (PCG), and p-cresol sulphate (PCS), particularly those bound to proteins, challenge conventional hemodialysis removal, contributing to CKD’s severity [4]. Disruption in the intestinal barrier and microbiota imbalance in CKD patients facilitates the translocation of toxins and bacteria into the bloodstream, exacerbating inflammation and cardiovascular risks [2, 5]. Since dialysis shows limited efficacy in toxin clearance, modulating the gut microbiota to reduce toxin production is a crucial management strategy for CKD [6].

Probiotics have been posited to beneficially influence the gut microbiota, potentially offering therapeutic benefits for CKD patients by restoring gut homeostasis, reducing uremic toxins, and thereby alleviating the metabolic burden associated with CKD [7–10]. Certain strains have shown promise not only in restoring gut barrier integrity and modulating immune responses but also in directly influencing uremic toxin levels, which could decelerate renal dysfunction [11, 12]. However, despite these findings, the evidence regarding the efficacy of probiotics in managing CKD, particularly in terms of reducing serum concentrations of uremic toxins, remains nascent and somewhat equivocal [13, 14].

In this study, we aimed to clarify the impact of dietary probiotic intervention on CKD progression, focusing on the probiotic strain *Bifidobacterium longum* subsp. *longum* BL21. This strain was selected based on its documented benefits in mitigating endotoxemia-related inflammation and restoring intestinal barrier function [15, 16]. We hypothesized that BL21 might modulate key gut microbiota constituents, such as the abundance or proportion of certain gut bacterial genera, thereby influencing systemic uremic toxin levels. Thus, in addition to analyzing circulating uremic toxins, we also analyzed the gut microbial communities in a CKD rat model seeks to illuminate the link between BL21 and CKD pathogenesis, providing insights into gut microbiota's role and informing targeted probiotic therapies for CKD management.

## Materials and Methods

### Establishment of CKD Rat Model via 5/6 Nephrectomy Method

**Fig. 1:**
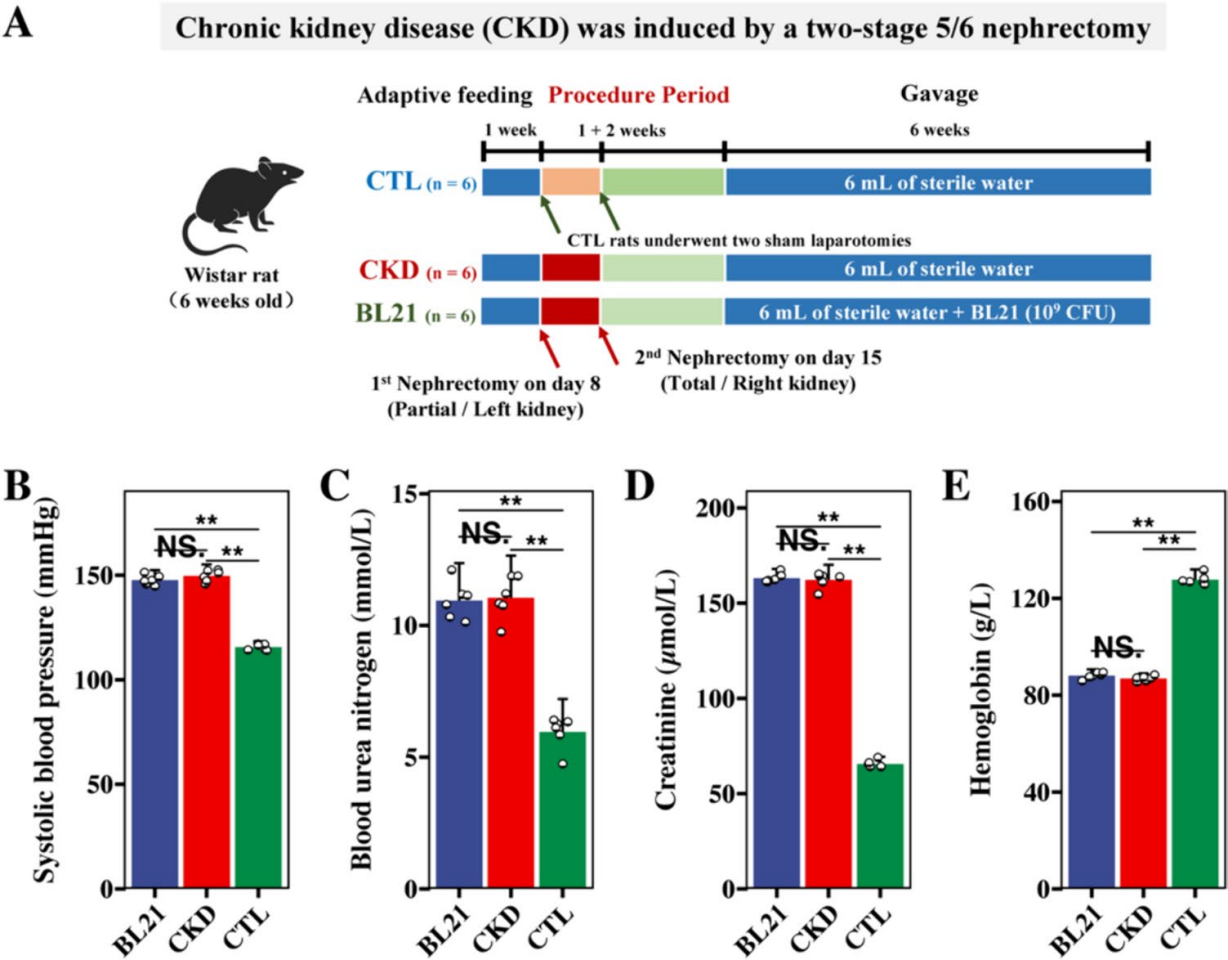
Overview of experimental procedures and baseline metrics in chronic kidney disease rats versus sham control. (**A**) outline of experimental procedures; B-E, comparative analysis of systolic blood pressure (**B**), blood urea nitrogen levels (**C**), creatinine concentrations (**D**), and hemoglobin values (**E**), across different rat groups, measured two weeks post the second stage of renal surgery. *, denotes *p* < 0.01; NS indicates no significant difference

Eighteen six-week-old male Wistar rats (200 $\pm$ 5 g) were procured from Shanghai Laboratory Animal Center. These rats were housed under controlled conditions, with a temperature maintained at 22 ± 2 °C and humidity at 55% ± 5%, adhering to a 12-h light/dark cycle. They had unrestricted access to food and water. All experimental procedures involving the rats conformed to the ethical guidelines for animal care and use as stipulated by the Shanghai Laboratory Animal Care and Animal Experimentation Center (license number 2022122003). The CKD rat model was developed using a 5/6 nephrectomy technique, as delineated in prior research [17]. This involved two-stage surgical procedures under anesthesia, either by excising approximately 5/6 of the renal mass (for the CKD and BL21 groups, n = 12) or performing a sham operation without nephrectomy (for the control [CTL] group, n = 6). Post-operatively, the rats were maintained in a specific pathogen-free environment and allowed ad libitum access to food and water. The establishment of renal failure was determined based on renal function assessments, overall animal health, and tail-cuff systolic blood pressure measurements. The CKD rat model was deemed successfully established two weeks following the second surgical procedure (as illustrated in Fig. 1A).

### Strain BL21 Preparation and Animal Experimentation

*B. longum* subsp. *longum* BL21 was obtained from Wecare Probiotics Co., Ltd. (Suzhou, China). The bacterium was cultivated in De Man, Rogosa, and Sharp (MRS) broth at 37 °C for a duration of 24 h [18]. Subsequently, the bacterial cells were harvested through centrifugation at 4,500 × *g* for 10 min and resuspended in sterile water to achieve a final concentration of 1 × 10^9^ CFU/mL.

Two weeks after the second surgical phase, the 12 CKD model rats were randomly allocated into two groups (n = 6 per group): the CKD group and the BL21 group. Throughout the experimental duration of six weeks, each rat received daily gavage based on the intervention assigned to their respective group. Specifically, rats in the CTL and CKD groups were administered 6 mL of sterile water each day. Conversely, the probiotic intervention group, designated as the BL21 group, received 6 mL of sterile water infused with *Bifidobacterium longum* subsp. *longum* BL21 at a concentration of 1 × 10^9^ CFU. Additionally, all groups were provided with standard chow and sterile water as detailed in Supplementary Table S1.

### Sample Collection and Analysis Procedures

During the course of the study, rats were accommodated in cages sterilized at high temperatures, facilitating natural defecation. Systematic collection of fecal samples was conducted on the initial day of each experimental week. Approximately 300 mg of feces were collected from each rat between 8 and 9 AM. These samples were secured in sterile 2 mL centrifuge tubes, immediately frozen with liquid nitrogen, and subsequently transported to the laboratory for preservation at -80 °C. For each rat, fecal samples were collected in two tubes per session. Blood sampling was executed two weeks post the second surgical phase and reiterated at the end of the experiment. Each rat contributed 1 to 2 mL of blood, collected between 10 and 11 am. post-centrifugation at 1200 × *g* for 10 min, 200 $\mu \text{L}$ of serum were extracted, aliquoted into 1.5 mL centrifuge tubes in eight separate portions, and stored at -80 °C.

Serum concentrations of various uremic toxins (IS [19], TMAO [20]), p-cresyl sulfate (PCS) [21], p-cresyl glucuronide (PCG) [21], and indole-3-acetic acid (IAA) [22] and lipopolysaccharides (LPS) were analyzed, being quantified using an enzyme-linked immunosorbent assay kit (Wuhan Chundu Biotechnology Co., Ltd.), adhering to the manufacturer's guidelines.

### Histological Analysis of Kidney and Colon

At the end of the 6-week intervention, rats were anesthetized and euthanized. Kidneys and colons were immediately removed, fixed in 10% formalin, and embedded in paraffin. Sections of 5 µm thickness were prepared for histological examination. Kidney sections were stained with Hematoxylin and Eosin (H&E), and colon sections with Periodic Acid-Schiff (PAS), followed by evaluation under a light microscope.

### Fecal Microbiota DNA Extraction and 16S rRNA Gene Amplicon Sequencing

Microbial DNA extraction from the fecal samples was conducted as described previously [23]. The 16S rRNA gene V3–V4 region was amplified by polymerase chain reaction (PCR) using 341F (5′-CCTACGGGNGGCWGCAG-3′) and 805R (5′-GACTACHVGGGTATCTAATCC-3′) primers. The PCR conditions were as follows: denaturation at 95 °C for 3 min; 21 cycles of 94 °C for 0.5 min, annealing at 58 °C for 0.5 min, and extension at 72 °C for 0.5 min; followed by a final extension at 72 °C for 5 min. The PCR products from different samples were indexed and mixed in equal ratios for sequencing, which was performed using the Illumina MiSeq platform (2 × 300 bp) according to the manufacturer’s instructions.

### Bioinformatics Analysis of 16S rRNA Gene Amplicons

Sequencing read pairs were demultiplexed based on unique molecular barcodes, and reads were merged using USEARCH Version 8.0. Merging revealed 0 mismatches and a minimum overlap of 50 bases. Sequences that could not be spliced and chimeras were removed using UCHIME software. Sequences shorter than 400 bases after splicing were removed. Operational taxonomy units were clustered using UPARSE software [24] (version 7.1 http://drive5.com/uparse/) based on 97% similarity. The phylogenetic affiliation of each 16S rRNA gene sequence was analyzed using RDP Classifier (http://rdp.cme.msu.edu/) against the RDP database (RDP Release 11) using a confidence threshold of 70%. Other analyses were performed using the QIIME 1.9 pipeline [25].

### Statistical Analysis

The nonparametric Kruskal–Wallis test was used for multiple group comparisons. The nonparametric Mann–Whitney *U* test was used to test for significant differences between two groups. *P* values < 0.05 were considered significant. Continuous data were expressed as means ± standard deviations. Principal coordinate analysis (PCoA) ordination plots for the gut microbiota-related parameters were constructed based on unweighted UniFrac distances, and significant differences between the CKD and CTL groups were determined using the *adonis2* function of the vegan package in R [26]. Linear discriminant analysis effect size (LEfSe) analysis was performed online using the microeco package [27]. Statistics and graphs for the gut microbiota-related parameters were generated using the ggplot2 package in R [28]. Spearman’s rank correlation test was performed to analyze the correlations between the significant serum uremic toxins and the significant fecal bacterial genera. All of the statistical analyses were performed using R 4.2.2.

## Results

### Establishment and Baseline Assessment of the CKD Rat Model Before BL21 Intervention

We constructed the CKD rat model using a two-stage 5/6 nephrectomy method (Fig. 1A). Two weeks post the second surgery, we performed baseline evaluations to confirm the CKD model's successful establishment. At this point, prior to BL21 intervention, the blood pressure and serological parameters of each operated rat were examined. Figure 1B–E illustrated that, when compared with the CTL (sham) group, both the BL21 and CKD groups exhibited significantly increased systolic blood pressure and elevated serum concentrations of blood urea nitrogen and creatinine, along with significantly decreased hemoglobin concentrations. Importantly, at this baseline phase, these were no significant differences between the BL21 and CKD groups in these measures. The observed elevations in systolic blood pressure and serum concentrations of blood urea nitrogen and creatinine, alongside reduced hemoglobin levels, are typical characteristics of CKD. This indicates the successful establishment of our CKD rat model, confirming that the model was appropriately suited for subsequent investigation of BL21 intervention.

### Effects of BL21 Intervention on Serum Uremic Toxins, Health, and Kidney Pathology in CKD Rats

**Fig. 2:**
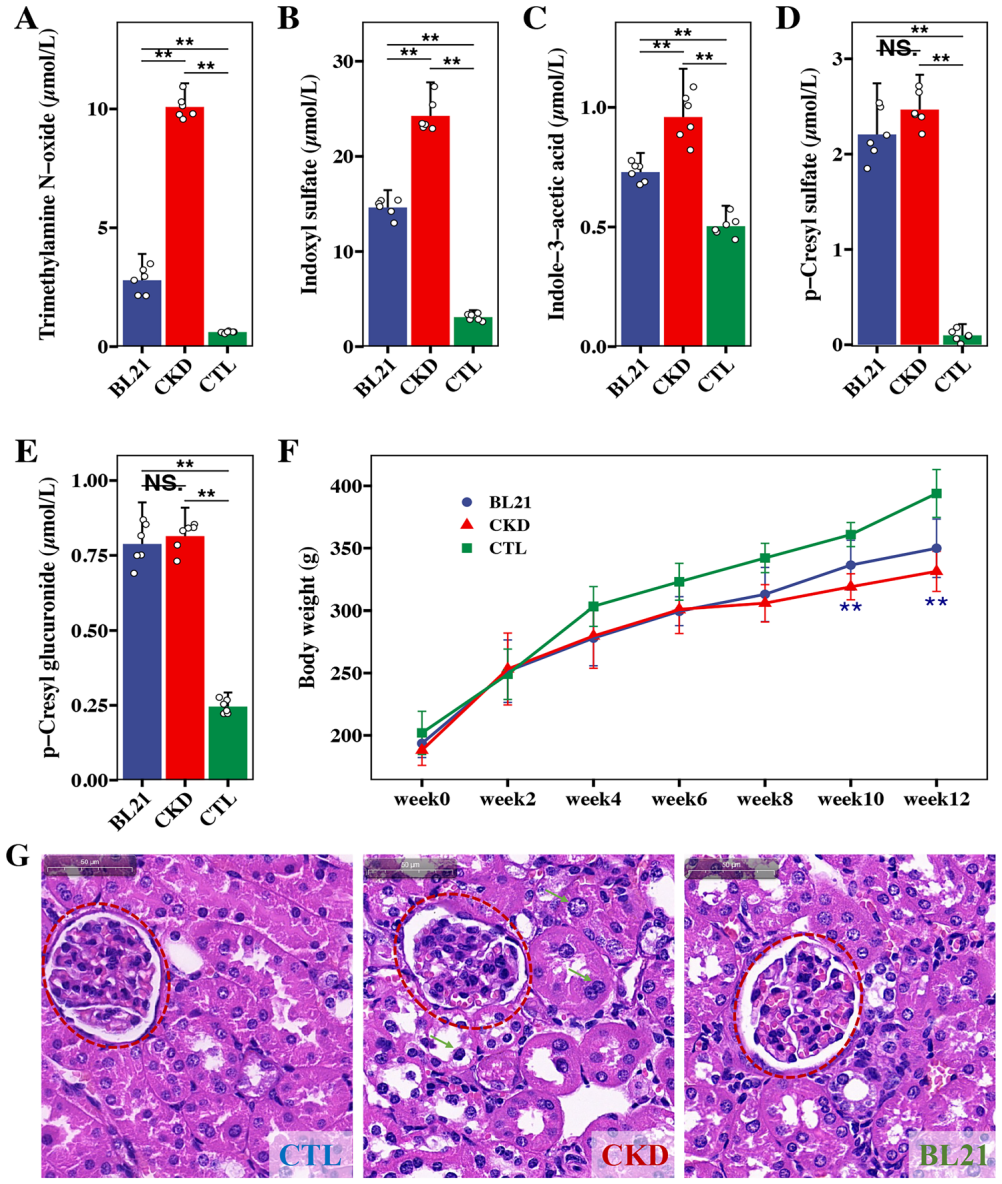
Impact of *Bifidobacterium longum* subsp. *longum* BL21 on chronic kidney disease rats. (**A**-**E**) reduction in serum concentrations of uremic toxins following dietary intervention with BL21. (**F**) increase in body weight of CKD rats’ post-intervention. (**G**) Hematoxylin and Eosin (HE) staining of kidney tissue. Compared to the CTL group, ** indicates *p* < 0.01; NS. denotes not significant

At the end of the intervention, a significant increase in serum uremic toxin concentrations was observed in both the CKD and BL21 groups when compared to the CTL group (*p* < 0.01, Fig. 2A-E). This finding underscores the severe impairment of renal function and the consequent accumulation of uremic toxins in CKD rat model. Notably, however, the BL21 intervention resulted in a marked reduction in serum levels of TMAO, IAA, and IS in comparison to the CKD group (*p* < 0.01, Fig. 2A-C). While the serum concentrations of PCS and PCG did not show a significant decrease in the BL21 group relative to the CKD group (Fig. 2D-E), a downward trend was evident. These observations collectively suggest that BL21 intervention has a beneficial effect in mitigating renal function impairment and reducing the accumulation of certain uremic toxins in CKD rats, highlighting its potential therapeutic role in CKD management.

In addition, rats in the CTL group were in good condition, moved flexibly, had normal stools, and showed no significant changes in food and water intake. In contrast, rats in the CKD group showed listlessness, decreased activity, and loss of appetite. Following BL21 intervention, these rats exhibited improved vitality and activity levels, more closely resembling the CTL group than the CKD group. Specifically, the BL21 group rats maintained regular food and water intake, showed normal stool consistency, and presented no significant signs of lethargy or appetite loss, which marks a notable improvement from the CKD condition. During the experiment, the CTL group rats exhibited a natural progression of weight gain. In comparison, both the CKD and BL21 groups showed a deceleration in weight increase, with the CKD group being particularly affected. After the eighth week, the CKD group’s weight gain slowed considerably, displaying a statistically significant difference from the CTL group (*p* < 0.01). Notably, compared with CKD group, following the intervention of BL21, there was a discernible trend toward normalization in weight gain, especially noticeable after two weeks of intervention (*p* < 0.01). The rats in the BL21 group began to show a recovery in their growth trajectory, aligning more closely with the pattern observed in the CTL group, as depicted in Fig. 2F.

In the renal histopathological evaluation, distinct pathological alterations were observed in the CKD group as compared to the CTL group (Fig. 2G). These alterations, emblematic of CKD, encompassed glomerular damage, interstitial fibrosis, and pronounced inflammatory cell infiltration. Contrastingly, post-intervention with BL21, a marked amelioration in renal histopathology was evident in the CKD cohort. This improvement was manifested by attenuated glomerular injury, reduced fibrosis, and a mitigated inflammatory response. These findings collectively indicate that BL21 intervention may play a therapeutic role in alleviating renal damage inherent to chronic kidney disease.

### Effect of BL21 Intervention on Intestinal Barrier Integrity in CKD Rats

**Fig. 3:**
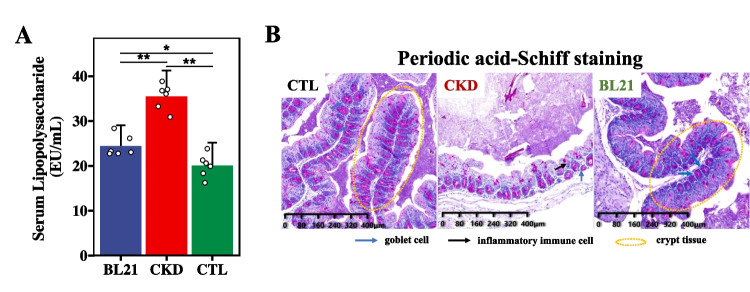
*Bifidobacterium longum* subsp. *longum* BL21 intervention effects on serum lipopolysaccharide (LPS) levels and intestinal barrier in CKD rats. (**A**) serum LPS concentration changes post-BL21 intervention; ** signifies *p* < 0.01; * represents *p* < 0.05. (**B**) Periodic acid-Schiff staining (PAS) of colon tissue, illustrating the impact on intestinal barrier integrity

In contrast with the CTL group, a significant elevation in serum LPS levels was observed in the CKD group (*p* < 0.01, Fig. 3A), possibly indicative of increased intestinal permeability and bacterial translocation. Compared to the CKD group, the BL21 intervention led to a significant reduction in serum LPS levels (*p* < 0.01, Fig. 3A), suggesting an enhancement in intestinal barrier function. It should be noted, though, that while the serum LPS levels in the BL21 group showed improvement, they did not fully reach the baseline levels observed in the CTL group. Considering the elevated serum LPS concentrations are associated with disruption of the intestinal mucosal barrier, we further investigated structural changes in the colon. Histological analysis of the colon using PAS staining revealed compromised mucosal integrity in CKD rats, characterized by disrupted crypt architecture, reduced goblet cell numbers, and increased infiltration of inflammatory immune cells, compared to the CTL group. Notably, BL21 intervention mitigated colonic inflammation and augmented goblet cell populations, thereby restoring intestinal barrier integrity (Fig. 3B).

### Effect of BL21 Intervention on Gut Microbiome Alterations in CKD Rats

**Fig. 4:**
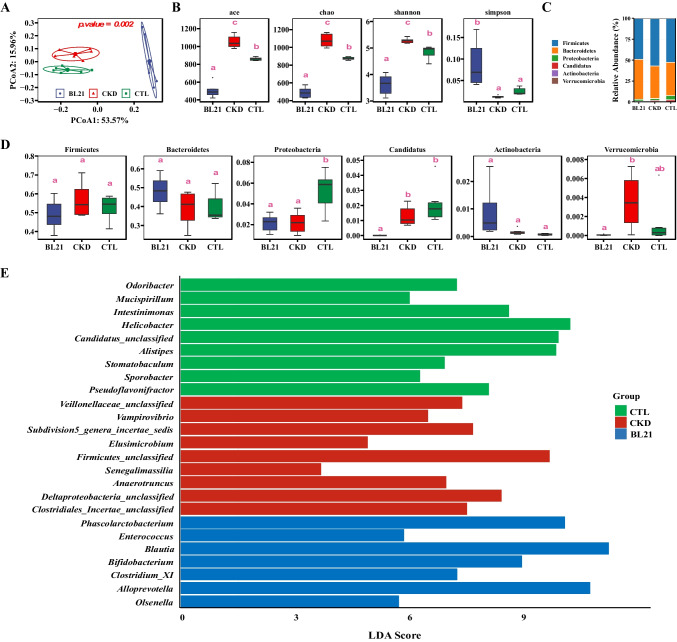
*Bifidobacterium longum* subsp. *longum* BL21 intervention impact on gut microbiota composition in chronic kidney disease (CKD) rats. (**A**) beta diversity alterations in gut microbiota due to BL21 intervention, as shown by Unweighted UniFrac Principal Coordinates Analysis (PCoA) of fecal samples, highlighting significant group differences after 6 weeks of intervention. (**B**) alpha diversity changes in gut microbiota among CKD rats post-BL21 treatment. (**C**–**D**) shifts in gut microbiota structure at the phylum levelwith. Different superscript letters indicate statistically significant differences (p < 0.05). (**E**) exploration of genus-level relative abundance changes in gut microbiota through Linear Discriminant Analysis Effect Size (LEfSe) analysis

This study explored the impact of BL21 on the gut microbiome of CKD rats. Beta diversity analysis based on phylogenetic distance revealed clustering of samples according to the intervention measures (Fig. 4A). Unweighted UniFrac PCoA demonstrated that a 6-week intervention with BL21 significantly affected the gut microbiota of CKD rats compared to the CTL group (Fig. 4A). Alpha diversity analysis indicated that, compared to the CTL group, the gut microbiome richness (ACE and Chao 1 indices) and diversity (Shannon index) in the CKD group were significantly increased (Fig. 4B). Following the intervention with probiotic BL21, there was a significant decrease in the ACE, Chao1, and Shannon indices of the gut microbiome, while the Simpson index significantly increased (Fig. 4B). At the phylum level, the gut microbiota of all three groups was predominantly composed of Firmicutes, Bacteroidetes, and Proteobacteria, accounting for over 98% of the total abundance (Fig. 4C). Although the relative abundance of Firmicutes tended to be higher and Bacteroidetes lower in the CKD group compared to other groups, there were no significant differences in the relative abundances of Firmicutes, Bacteroidetes, and Actinobacteria among the three groups (Fig. 4D). The relative abundance of Proteobacteria was significantly lower in the CKD and BL21 groups compared to the CTL group. Additionally, compared to the CKD group, the relative abundance of Verrucomicrobia in the BL21 group was significantly reduced. Genus-level LEfSe analysis revealed intergroup differences in the abundance of specific bacterial genera (Fig. 4F). In the CKD group, genera such as *Vampirovibrio*, *Deltaproteobacteria _ unclassified*, and *Clostridiales _ Incertae _ unclassified* were significantly enriched, while the abundance of genera such as *Odoribacter*, *Mucispirillum*, and *Alistipes* was significantly reduced. However, after supplementation with BL21, there was a significant enrichment of beneficial bacterial genera such as *Bifidobacterium*, *Phascolarctobacterium*, and *Blautia*. This enrichment may suggest the potential of BL21 for colonization in the gut, although further studies are required to confirm this hypothesis.

### Correlation Between Microbial Genera and Uremic Toxins in CKD Rats

**Fig. 5:**
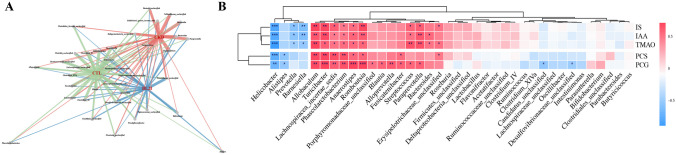
Correlation analysis between microbial genera and uremic toxins in CKD rats. (**A**) Network diagram constructed based on significant differences (*p* < 0.05). Nodes (circles) represent microbial genera, with edges indicating significant differences between specific genera and groups. The thickness of the edges corresponds to the level of significance, with thicker lines denoting greater significance. (**B**) Correlation between serum concentrations of uremic toxins (indoxyl sulfate [IS], indole-3-acetic acid [IAA], trimethylamine oxide [TMAO], p-cresol sulphate [PCS], and p-cresol glucuronide [PCG]) and gut microbiota. Bars adjacent to the dendrogram are color-coded to reflect significant correlations

To further investigate the influence of specific alterations in the intestinal microbiome on uremic toxin levels in CKD rats, this study performed an in-depth statistical analysis of the gut microbiota at the genus level across different groups. The findings are presented in Fig. 5A and Table S2. The network diagram in Fig. 5A illustrates the species exhibiting statistically significant differences (*p* < 0.05) among the CTL, CKD, and BL21 groups. Each node in the diagram represents a genus, with the thickness of the edges between nodes indicating the degree of significance (the thicker, the more significant). Relative to the CTL group, the CKD group exhibited notable changes in the relative abundance of 10 genera (as detailed in Fig. 5A and Table S2), including significant increases in *Bacteroides*, *Phascolarctobacterium*, *Paraprevotella*, *Veillonellaceae_unclassified*, *Prevotellaceae_unclassified*, and *Elusimicrobium*. Conversely, there was a significant reduction in the relative abundance of genera such as *Barnesiella*, *Helicobacter*, and *Alistipes*, suggesting substantial alterations in the intestinal microbiota composition, potentially indicative of CKD-associated microbial dysbiosis. In comparison with the CKD group, BL21 intervention led to significant shifts in 25 genera (refer to Fig. 5A and Table S2), characterized by a marked increase in the relative abundance of beneficial bacteria such as *Bifidobacterium*, *Barnesiella*, *Blautia*, *Alloprevotella*, *Phascolarctobacterium*, *Helicobacter*, and a decrease in *Lachnospiraceae_unclassified*, *Ruminococcaceae_unclassified*, and *Bacteroides*, among others. Notably, BL21 intervention significantly counteracted the CKD-induced reduction in the abundance of genera *Barnesiella* and *Helicobacter*, which exhibited an inverse correlation with serum uremic toxin concentrations (as shown in Fig. 5B, Table S3). In contrast, the abundance of *Bacteroides* and *Paraprevotella*, which increased in the CKD group and positively correlated with serum uremic toxin concentration, was significantly reversed following BL21 intervention. Additionally, BL21 intervention diminished the abundance of *Veillonellaceae_unclassified* and *Elusimicrobium*, which were elevated in the CKD group. The observed significant increase in the relative abundance of *Bifidobacterium* in the gut microbiota post BL21 supplementation indicates successful colonization and potential probiotic activity of the strain BL21. However, despite the increased abundance of *Bifidobacterium*, no direct correlation with uremic toxin levels was observed in Fig. 5B. These results suggest that BL21 intervention effectively modulates the alterations and imbalances in CKD-related intestinal microbiota composition, characterized by an increase in specific beneficial bacterial genera and a decrease in potentially harmful ones. This indicates that BL21, as a probiotic intervention, may effectively modulate the gut microbial community structure, offering potential efficacy in managing CKD-associated gut dysbiosis.

## Discussion

The gut-renal axis is increasingly recognized as a novel therapeutic target in CKD management [29]. In CKD patients, a notable shift in the gut microbiota composition occurs, largely driven by an overgrowth of protein-degrading bacteria such as Actinomycetes, Proteobacteria, and Firmicutes, a process exacerbated by elevated urea levels, a hallmark of uremic retention. This microbial imbalance enhances intestinal wall permeability, potentially facilitating the translocation of bacteria or their components into the bloodstream, which can precipitate systemic inflammation and atherosclerosis. Probiotics, by selectively promoting the growth and/or activity of host-beneficial bacteria, offer a promising approach to reconstitute the gut microbiota in CKD patients [2, 30–32]. This study aimed to assess the impact of oral probiotic BL21 intervention on uremic toxin levels, growth status, intestinal barrier function, and alterations in the gut microbiota composition in a CKD rat model, thereby elucidating BL21’s potential mechanisms in CKD management. Our findings revealed that CKD rats exhibited significantly higher levels of serum uremic toxin and LPS, which correlated with overall health decline, renal histopathological alterations, compromised intestinal barrier function, inflammatory responses, and disrupted intestinal microbiota. These observations align with those reported in CKD patients [33, 34]. Notably, treatment with BL21 partially relieved these CKD-related symptoms, including severe renal impairment and accumulation of uremic toxins. Furthermore, BL21 markedly decreased serum LPS levels, suggesting an enhancement in intestinal barrier integrity. Given that LPS is a known indicator of increased intestinal permeability and bacterial translocation, its association with intestinal barrier damage is evident in various disease states, including CKD [35]. The reduction in serum LPS levels by BL21 potentially aids in mitigating intestinal barrier dysfunction, thereby curtailing the translocation of bacterial products and the consequent inflammatory responses. Additionally, our observations of diminished colon inflammation and an increase in goblet cells in colon tissues further corroborate the therapeutic benefits of BL21. However, despite BL21’s regulatory influence on uremic toxin levels, these levels remained elevated compared to normal, highlighting the complexity of uremic toxin dynamics in CKD [36]. This complexity might be influenced by variables such as the dosage, combination, or duration of probiotic intervention. Consequently, a continuous intake of probiotic BL21 might be essential to sustain the therapeutic effects of BL21. Considering the potential transient nature of probiotic colonization, future research should investigate the long-term effects on CKD symptoms following the discontinuation of probiotic BL21 intake, as well as the sustained impact of BL21 on the stability of the intestinal microbial community. These studies are crucial for understanding the persistence and optimal application strategies of BL21 interventions.

In our study, we discerned a pronounced augmentation in the richness and diversity of the intestinal microbiota in CKD conditions, likely reflecting changes in the intestinal environment due to renal dysfunction. These alterations may precipitate a disproportionate proliferation of specific bacterial genera, culminating in a disruption of the equilibrium within the gut microbial ecosystem. This dysbiosis is linked to several health issues, including impaired intestinal barrier function and systemic inflammation [37]. Following BL21 treatment, we noted a considerable decrease in the ACE, Chao1, and Shannon indices, indicating a reduction in microbial species richness due to the decline of specific bacterial taxa. Concurrently, the Simpson index increased, suggesting a greater dominance of certain microbial species. This pattern, likely a result of BL21’s influence on the intestinal environment, mirrors the alpha diversity trends seen in BL21’s use against type 2 diabetes and alcoholic liver disease [18, 38]. Therefore, despite an overall decrease in diversity, this suggests a beneficial realignment of the gut microbiome. In the context of CKD’s typical microbiota imbalance, the probiotic’s 'streamlining' effect could be advantageous, reducing pathogenic bacteria overgrowth and promoting beneficial microbial balance, crucial for restoring intestinal barrier integrity and overall health. Thus, BL21’s strategic use is key in optimizing the gut microbiota, highlighting the importance of understanding microbiota dynamics in disease states.

Microbiome genomics analysis in CKD revealed a key phenomenon, a decrease in bacteria possessing enzymes for short-chain fatty acids (SCFAs) production, and an increase in those with urease, uricase, p-cresol, and indole-forming enzymes [39, 40]. This shift may lead to reduced production of beneficial micronutrients and increased generation of toxic solutes, potentially accelerating CKD progression [41]. CKD, characterized by gradual loss of renal function, affects various metabolic functions of the kidneys, including waste excretion, electrolyte balance, blood pressure regulation, and erythropoiesis [37]. Its development is associated with abnormalities in multiple metabolic pathways, including glucose and lipid metabolism, mineral and bone metabolism. BL21 plays a crucial role in regulating oxidative-antioxidative reactions, the immune system, and glucose and lipid metabolism [15, 18]. SCFAs like butyrate, propionate, and acetate, produced by gut microbiota through the fermentation of complex carbohydrates, are vital for maintaining intestinal health and regulating systemic metabolism. In our study, BL21 intervention significantly increased the abundance of *Barnesiella*, *Helicobacter*, and *Bifidobacterium*. *Barnesiella* is implicated in the breakdown of cellulose and other complex carbohydrates, modulating the gut environment and immune responses, thereby promoting SCFAs production [42]. Similarly, certain species of Helicobacter are considered to play a role in regulating the gut environment and immune responses [43, 44]. *Bifidobacterium* is recognized as beneficial due to its close association with dietary fiber metabolism and inflammation suppression [15]. Therefore, the therapeutic effects of BL21 we observed may relate to the increased abundance of these beneficial genera, particularly in their role in enhancing SCFAs production. SCFAs are not only crucial for intestinal health but also influence systemic metabolism and immune functions through various mechanisms, potentially improving CKD’s clinical symptoms and pathological processes [45]. These findings suggest that modulating the gut microbiota with probiotics could be an effective strategy to complement dialysis treatment. Several clinical trials have tested probiotics in CKD treatment [34], mostly involving *Lactobacillus* and *Bifidobacterium*, with reported benefits including reduced urea, blood urea nitrogen, and ammonia levels, as well as decreased plasma concentrations of PCS and IS. However, more extensive preclinical animal studies and mechanistic research are needed for the effective clinical application of probiotics in CKD management.

**Fig. 6:**
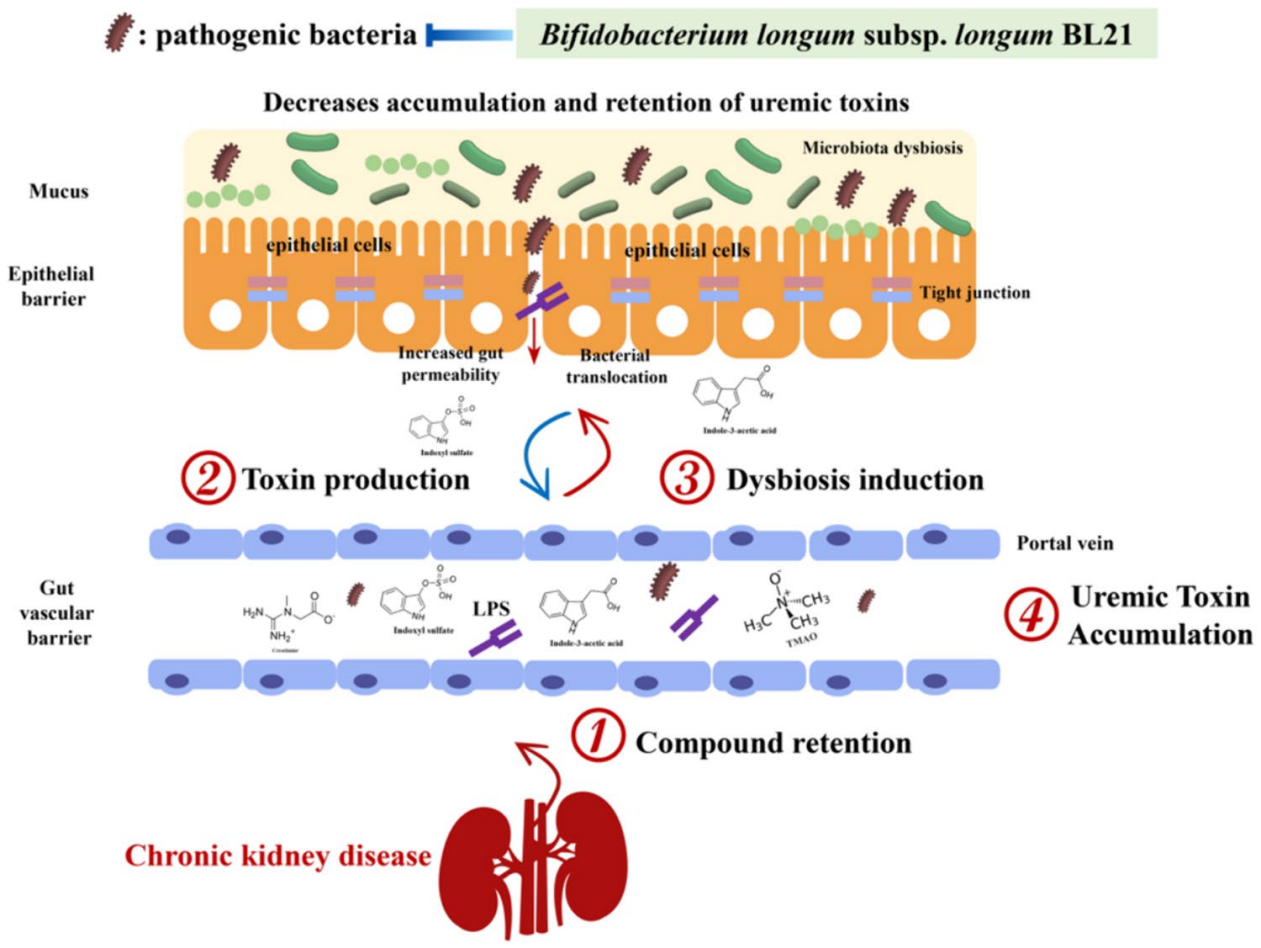
*Bifidobacterium longum* subsp. *longum* BL21 ameliorates the symptoms of chronic kidney disease by modulating the gut microbiota

Through our analysis, we noted a significant association between certain gut microbial genera and the production of uremic toxins, whereas other genera did not demonstrate this correlation [46, 47]. This finding prompted deeper contemplation on the complex interplay between gut microbial diversity and CKD pathology. For instance, CKD rats exhibited significant alterations in their gut microbiota compared to CTL rats. Notably, the substantial increase in *Bacteroides* and *Paraprevotella*, along with the decrease in *Helicobacter* and *Barnesiella*, closely correlated with changes in serum uremic toxin levels. *Bacteroides* and *Paraprevotella* were positively correlated with uremic toxin concentrations, whereas *Helicobacter* and *Barnesiella* were negatively correlated. These findings suggest that BL21 effectively alleviates the disease progression in CKD rats by modulating gut bacteria associated with uremic toxins. The benefits of BL21 may relate to reduced inflammation and improved gut microbiota homeostasis, particularly by increasing the abundance of genera like *Helicobacter*, *Barnesiella*, and *Bifidobacterium*, which help maintain intestinal barrier integrity. This is crucial in CKD, as increased gut permeability may exacerbate uremic toxin accumulation and systemic inflammation [35]. Additionally, BL21 intervention significantly lowered serum LPS levels, further confirming its positive impact on intestinal barrier function. Figure 6 illustrates the influence of BL21 intervention on gut microbiota composition and its correlation with reduced serum uremic toxin concentrations, highlighting BL21’s potential role in regulating gut microbial community balance, maintaining intestinal barrier integrity, and reducing inflammation and uremic toxin accumulation.

Overall, BL21 intervention, by modulating specific gut bacterial species, improved gut microbiota homeostasis, thereby protecting renal function in CKD rats. Our results reveal that changes associated with probiotics in CKD partly stem from alterations in the gut microbiota, affecting uremic toxin dynamics and improving intestinal epithelial barrier integrity, thereby reducing inflammation. However, the complete mechanism linking renal function and gut microbiota remains to be further elucidated. A limitation of our study is the small sample size in animal experiments, which may lead to data variability. Additionally, since CKD rats still retain residual renal function, serum TMAO levels may not fully represent intestinal trimethylamine concentrations. Future research needs to clarify the pathophysiological roles of gut bacteria producing uremic toxins, such as their impact on intestinal barrier function and inflammation, to provide theoretical support for developing effective probiotic interventions for CKD management.

## Conclusion

Our study suggests the intestinal microbiome is a promising target for CKD management. The results of animal experiments showed that *B. longum* subsp. *longum* BL21 significantly modulated the gut microbiota and reduced serum concentrations of uremic toxins in a CKD rat model. Therefore, our findings provide evidence to facilitate future investigations of the relationships between diets, gut microbiota, and CKD, and future applications of dietary probiotic interventions for the prevention and treatment of CKD and its associated metabolic disorders.

## Supplementary Information

Below is the link to the electronic supplementary material.

Supplementary file1 (XLSX 23 KB)

## Data Availability

The 16S rRNA gene sequence of rat fecal has been submitted to NCBI genome database under PRJNA995383.
